# Role of 2D and 3D defects on the reduction of LaNiO_3_ nanoparticles for catalysis

**DOI:** 10.1038/s41598-017-10703-5

**Published:** 2017-08-30

**Authors:** Sarika Singh, Eric Prestat, Liang-Feng Huang, James M. Rondinelli, Sarah J. Haigh, Brian A. Rosen

**Affiliations:** 1 Department of Materials Science and Engineering, Tel Aviv University, 55 Haim Levanon Street, Ramat Aviv, 69987001 Israel; 2 School of Materials, The University of Manchester, Oxford Road, Manchester, M13 9PL UK; 3 Department of Materials Science and Engineering, Northwestern University, Evanston Illinois, 60208-3108 USA

## Abstract

Solid phase crystallization offers an attractive route to synthesize Ni nanoparticles on a La_2_O_3_ support. These materials have shown great promise as catalysts for methane oxidation and similar reactions. Synthesis is achieved by the reduction of a LaNiO_3_ (LNO) precursor at high temperatures, but the reduction pathway can follow a variety of routes. Optimization of catalytic properties such as the long-term stability has been held back by a lack of understanding of the factors impacting the reduction pathway, and its strong influence on the structure of the resulting Ni/La_2_O_3_ catalyst. Here we show the first evidence of the importance of extended structural defects in the LNO precursor material (2D stacking faults and 3D inclusions) for determining the reaction pathway and therefore the properties of the final catalyst. Here we compare the crystallization of LNO nanoparticles via two different pathways using *in-situ* STEM, *in-situ* synchrotron XRD, and DFT electronic structure calculations. Control of extended defects is shown to be a key microstructure component for improving catalyst lifetimes.

## Introduction

Lanthanum nickelate perovskites (LaNiO_3_) have attracted considerable interest in the past decade, not least because of their use as a precursor to form Ni nanoparticle catalysts supported on La_2_O_3_ (Ni/La_2_O_3_)^1–7^. When LaNiO_3_ is heated in a reducing environment Ni atoms are exsolved in a process known as solid-phase crystallization^8–10^. Control of this reaction is key to optimizing the properties of the resulting catalytic material, yet the reaction mechanism and role of structural defects is still poorly understood. Here, we report the first direct work to demonstrate the effect that extended defects in the LaNiO_3_ precursor have on the reduction pathway, the strength of the bonding between the resulting Ni catalyst and its La_2_O_3_ support, and the ultimate performance of the Ni/La_2_O_3_ catalyst under methane reforming conditions.

Ni/La_2_O_3_ has the potential to catalyze methane oxidation and similar reactions while possibly resisting common mechanisms that drive catalyst deactivation such as carbon accumulation, sintering, and oxidation^11^. Solid-phase crystallization has therefore become a highly studied method for producing catalysts to service the gas reforming industry. The ability of Ni/La_2_O_3_ to show stable catalytic performance is related to the dispersion of Ni on the La_2_O_3_ support and the strength of the metal bond with the oxide support. These parameters have been previously reported to depend on the LaNiO_3_ synthesis method, but there is no clear understanding about how to predict which pathway will be taken and why one route may be preferable from a catalytic performance perspective^12, 13^. The two most reported reaction pathways are: a 3-step pathway where LaNiO_3_ is reduced through A_n+1_B_n_O_3n+1_ Ruddlesden-Popper (RP) phases (Reactions 1–3); and a 2-step pathway where LaNiO_3_ is reduced through a A_n_B_n_O_2n+1_ Brownmillerite phase (Reactions 4–5)

Three-step reduction of LaNiO_3_ through RP phases

1 $$4{{\rm{LaNiO}}}_{3}+2{{\rm{H}}}_{2}\to {{\rm{La}}}_{4}{{\rm{Ni}}}_{3}{{\rm{O}}}_{10}+{{\rm{Ni}}}^{0}+2{{\rm{H}}}_{2}{\rm{O}}$$

2 $${{\rm{La}}}_{4}{{\rm{Ni}}}_{3}{{\rm{O}}}_{10}+3{{\rm{H}}}_{2}\to {{\rm{La}}}_{2}{{\rm{NiO}}}_{4}+2{{\rm{Ni}}}^{0}+3{{\rm{H}}}_{2}{\rm{O}}$$

3 $${{\rm{La}}}_{2}{{\rm{NiO}}}_{4}+{{\rm{H}}}_{2}\to {{\rm{Ni}}}^{0}+{{\rm{La}}}_{2}{{\rm{O}}}_{3}+{{\rm{H}}}_{2}{\rm{O}}$$

Two-step reduction of LaNiO_3_ through brownmillerite phase

4 $$2{{\rm{L}}{\rm{a}}{\rm{N}}{\rm{i}}{\rm{O}}}_{3}+{{\rm{H}}}_{2}\to {{\rm{L}}{\rm{a}}}_{2}{{\rm{N}}{\rm{i}}}_{2}{{\rm{O}}}_{5}+{{\rm{H}}}_{2}{\rm{O}}$$

5 $${{\rm{La}}}_{2}{{\rm{Ni}}}_{2}{{\rm{O}}}_{5}+2{{\rm{H}}}_{2}\to 2{{\rm{Ni}}}^{0}+{{\rm{La}}}_{2}{{\rm{O}}}_{3}+2{{\rm{H}}}_{2}{\rm{O}}$$

Although both reaction pathways have been reported extensively in the literature, there has been little consideration of why one pathway may be preferred over another. LaNiO_3_ may be synthesized by a variety of methods including chemical co-precipitation^14, 15^, hydrothermal^14, 16^, sol-gel^17–21^, combustion^22, 23^, spray pyrolysis^16, 24^, and microwave synthesis^25^. However, there is no clear relationship between the synthesis method and reduction route (see Supporting information, Table S1).

This suggests that the factors influencing the reduction pathway are based on more subtle structural considerations such as the presence of extended defects or impurities in the precursor, a hitherto neglected factor that can strongly influence the crystallization mechanism and final catalyst properties. In this work LaNiO_3_ was synthesized using the chemical co-precipitation (hereafter written as cp-LNO) and hydrothermal methods (ht-LNO).

The average crystal structure of both materials is nearly identical as demonstrated by the similarity of the room temperature X-ray diffraction (XRD) patterns for both LaNiO_3_ products (Fig. S1). Most previous studies have determined the reaction pathway indirectly using temperature programmed reduction (TPR).

Here, we combine *in-situ* synchrotron-XRD and scanning transmission electron microscopy (STEM) with electron energy loss spectroscopy (EELS). Both characterization techniques were performed at elevated temperature (up to 800 °C) and in a reducing 3% H_2_/N_2_ gas atmosphere. This approach allows us to directly track the evolution of different crystal phases as well as correlate these to nanoscale measurement of local morphological and structural features. We reveal that our two precursor materials form Ni/La_2_O_3_ via different pathways, resulting in catalysts with differing stabilities.

**Figure 1:**
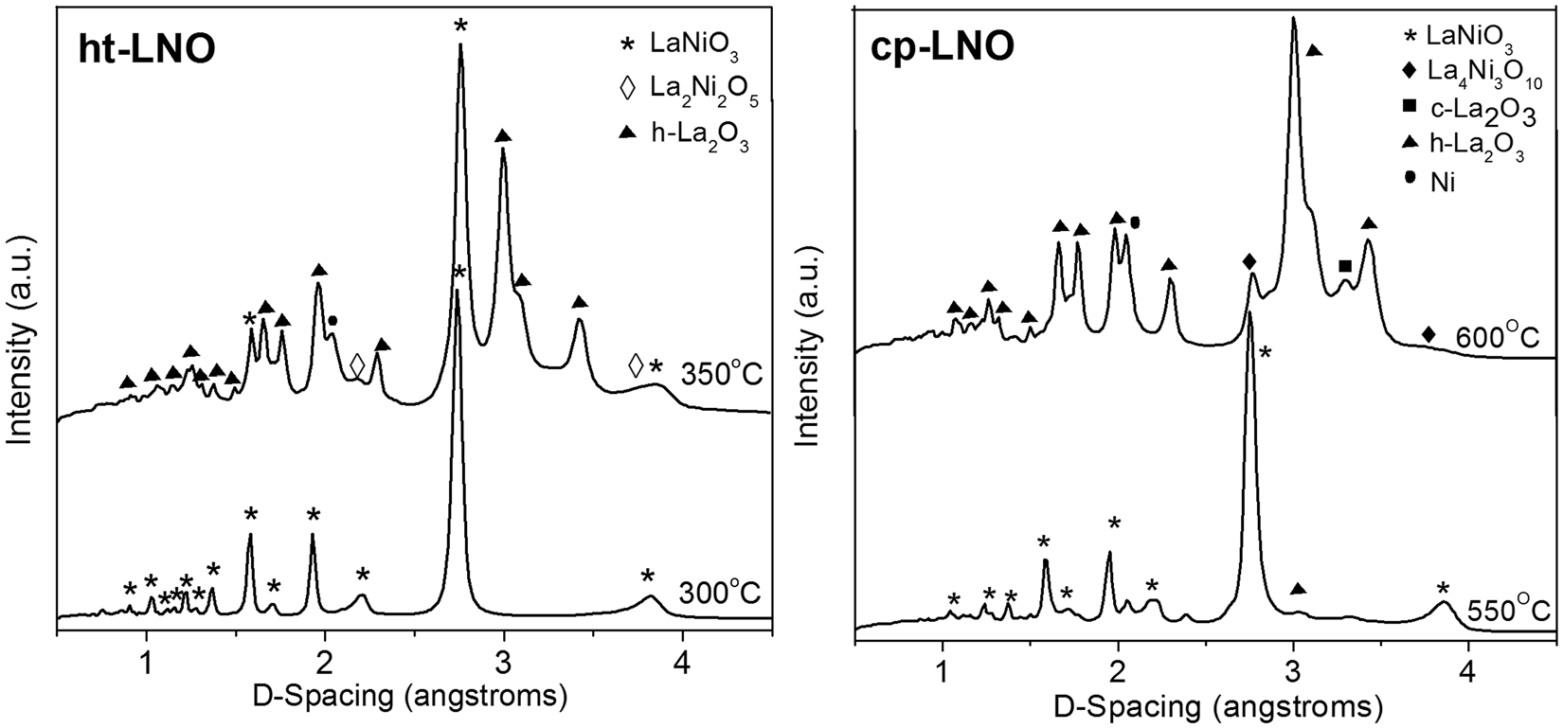
*In-situ* synchrotron XRD of ht-LNO (left) and cp-LNO (right) during reduction in 3%H_2_/N_2_. Data is shown at the critical temperature where the first intermediate is observed.

Synchrotron XRD is shown in Fig. 1. The results reveal that cp-LNO was reduced via the three-step pathway while ht-LNO was reduced via the two-step pathway. Key to identifying the pathways was the presence of the RP-phase, La_4_Ni_3_O_10_ (PDF#04-009-1774) or the Brownmillerite phase, La_2_Ni_2_O_5_ (PDF#04-016-0996). Here, we show the critical transition temperature where the first reduction intermediate in each case is first observed.

*In-situ* XRD suggests that cp-LNO may have a lower reducibility compared its ht-LNO counterpart, with the first reduced intermediate being observed at 550–600 °C compared to just 300–350 °C for ht-LNO. Furthermore, the temperature above which the original LNO perovskite structure was completely destroyed was 175 °C higher for cp-LNO compared to ht-LNO (Fig. S2). Interestingly both the hexagonal and cubic polymorphs of La_2_O_3_ (PDF#97-064-1603 and 00-02-0369 respectively) were formed during the 3-step process, although above 650 °C, only the hexagonal polymorph was observed. For ht-LNO only the hexagonal polymorph was observed during the entire reduction process (2-step reduction). TPR shown in Fig. S3 also characterizes the differences in reducibility of between cp-LNO ht-LNO, particularly at temperatures above 500 °C.

Since both precursor materials had similar average atomic structure, these differences in reduction pathway must be related to nanoscale structural and morphological features. (S)TEM is a highly effective means of characterizing structure and chemistry at the atomic scale, yet the ready oxidation of Ni nanoparticles in air makes *ex-situ* investigations of these materials challenging. *In-situ* environmental cell STEM studies are therefore attractive as they prevent unintended oxidation of the reduced species as well as allowing direct imaging (and more recently spectroscopic characterization) of a specimen region of interest during reduction^26^.

**Figure 2:**
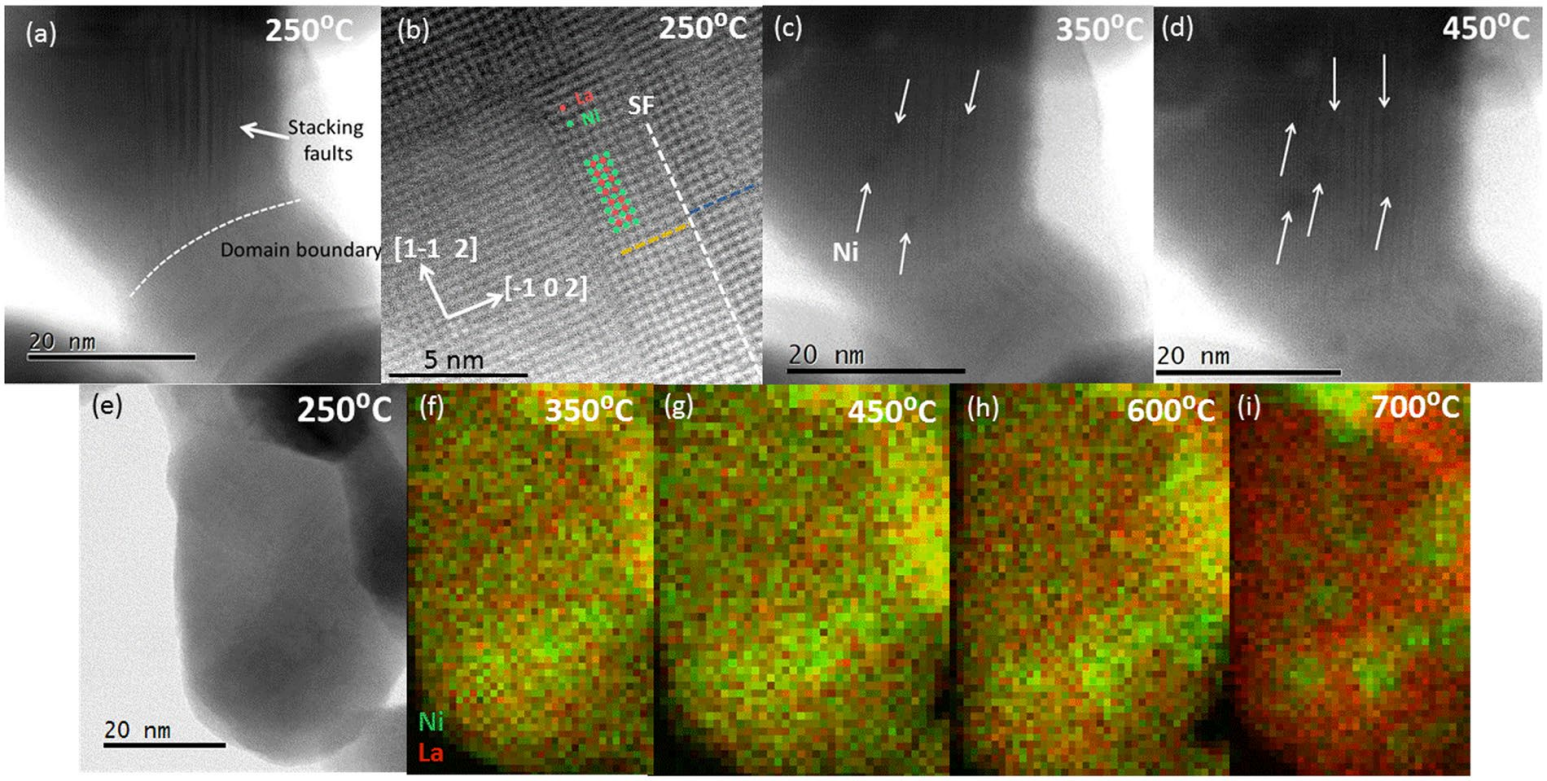
Bright-field STEM images of cp-LNO imaged in an environmental cell containing H_2_ gas at 250mbar at elevated temperature at (**a**,**b**) 250 °C; (**b**) high magnification of stacking fault showing ½ cell displacement characteristic of the RP-fault and overlay of Ni and La atomic positions, (**c**) 350 °C; and (**d**) 450 °C. Arrows in (**c**) and (**d**) indicate the locations of growing Ni crystals. (**e**) Bright field image of ht-LNO at 250 °C and EELS mapping of Ni (green) and La (red) in the ht-LNO sample at (**f**) 350 °C, (**g**) 450 °C, (**h**) 600 °C, and (**i**) 700 °C.

Bright field (BF) STEM micrographs of cp- and ht-LNO in the *in-situ* environmental cell at 250 °C before reduction (Fig. 2a,e) show that the only appreciable difference between the two LaNiO_3_ precursors is the type of defect present. In this study, 4 separate batches of each precursor were prepared, and over 1,000 images were analyzed by (S)TEM. The images shown in Fig. 2 and the defect structure of each precursor were found to be characteristic of the entire sample. The cp-LNO had a high concentration of 2D-defects in the form of stacking faults, while the ht-LNO was nearly free of this type of defect. The stacking faults here are similar to those reported in the LaCoO_3_ system in that they appear to originate from domain boundaries and that they come in bunches^27^. Such faults were not observed in the hydroxide precursors prior to their calcination to form the perovskite phase (Fig. S4). Hence, these stacking faults are expected to have been formed during the final calcination step of the cp-LNO synthesis where NiOH and LaOH were heated to 800 °C in an oxygen atmosphere.

BF STEM multislice simulations (Fig. S5) were conducted in order to understand the image contrast of LaNiO_3_. These calculations showed that the dark dots in the bright field STEM images are associated with La columns and the bright dots are associated with Ni columns. Pristine stacking in the [−1 0 2] direction should correspond to LaO-NiO_2_-LaO-NiO_2_ stacking. The dark contrast inside the stacking fault defect (Fig. 2a) as well as the half-period displacement of the La-columns across the stacking fault (Fig. 2b) are characteristic of a Ruddlesden-Popper (RP) fault due to the double LaO layer^28^.

In contrast, the ht-LNO was nearly free of the 2D stacking faults found in cp-LNO. However, EELS analysis of ht-LNO revealed small Ni-rich regions identified as NiO inclusions by their lattice spacing; these NiO inclusions can be thought of as 3D defects within LNO matrix^16^. In order to understand whether the different morphological defects impact reduction behavior, STEM imaging was performed while heating the cp- and ht-LNO samples to drive the solid-phase crystallization process. At 350 °C, 2–3 nanometer sized Ni particles were observed to nucleate in the region of the stacking faults of the cp-LNO particle (Fig. 2c). These Ni particles are associated with the Reaction 1. Peak broadening for nucleated particles below 10 nm obstructs their ready detection in XRD until they have grown larger at higher temperatures. This leads to some discrepancy between the XRD and STEM methodologies when determining key transformation temperatures.

Importantly, STEM imaging shows that the nucleation of Ni nanoparticles occurred only in the region of the stacking faults and not in the areas free of defects. This observation suggests that defects in the precursor phase influence the reduction pathway. We hypothesize that the stacking faults facilitate the nucleation of Ni particles by acting as paths for enhanced diffusion or by lowering the activation energy required for nucleation. When the temperature was increased to 450 °C, the Ni particles continued to grow while at the same time the stacking faults appeared to be dynamically changing (Fig. 2c,d). The apparent motion of the stacking faults can be explained by the diffusion of Ni ions adjacent to the fault and charge compensation by the creation of oxygen vacancies during the crystallization process^27^.

EELS analysis of the ht-LNO in a heated STEM cell revealed that, similar to the case of cp-LNO, the defect sites are key to determining the reduction pathway. The site of the NiO inclusions (Fig. 2f) were found to be the locale of Ni-enrichment during the reduction process, and ultimately the position of the metallic Ni nanoparticles at the end of the crystallization process (Fig. 2f–i). Individual EELS maps can be found in the Supporting information section (Fig. S6).

**Figure 3:**
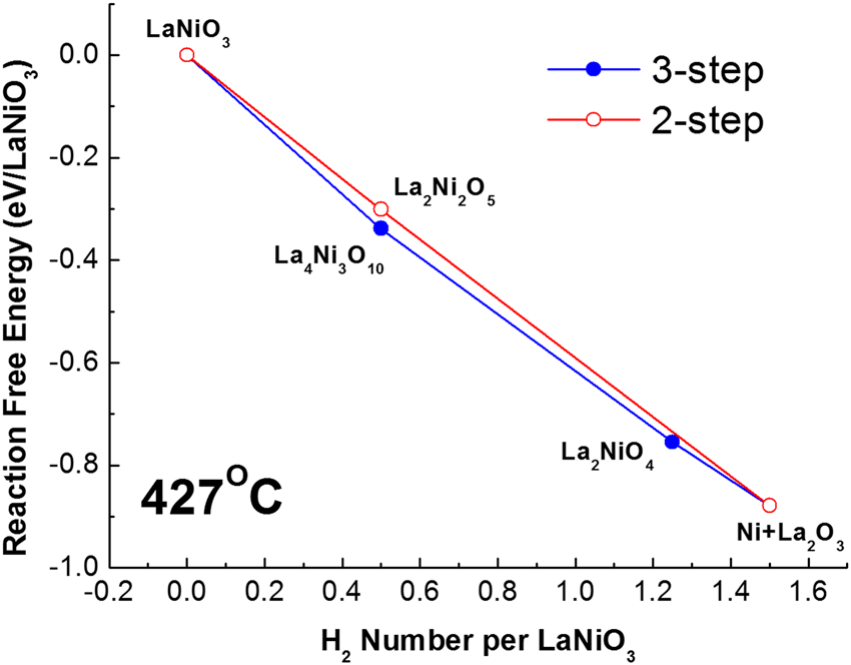
MS2 density functional theory calculation showing the reaction free energy for the 3- and 2-step crystallization paths.

Figure 3 shows the reaction free energies of each pathway, calculated with density-functional theory (DFT) simulations using the metaGGA MS2 exchange-correlation functional^29^ which yields remarkably accurate thermodynamic energies for various materials^30, 31^.

The free energies of each compound and their formulation are detailed in the Supporting information (Fig. S7). These DFT results show that the free energies of the intermediates for each pathway are similar. As a result, it is unlikely that the free energies of the intermediate states play a pivotal role in pathway selection. Instead, our *in-situ* XRD and STEM data suggests the reduction pathway is determined by the activation energy needed for nucleation of the different reduction intermediates. Defect sites in both materials (stacking faults in the cp-LNO and NiO inclusions in the ht-LNO) appear to be low energy nucleation sites for crystallization and hence the determining factor influencing the pathway. For example, stacking faults are likely influencing the material to favor the growth of layered RP- intermediates (via the 3-step mechanism) since the faults themselves are structurally similar to a RP phase. Similarly, NiO inclusions in ht-LNO appear to provide a low energy pathway for the nucleation of La_2_Ni_2_O_5_ (via the 2-step mechanism).

To test the catalytic stability of Ni/La_2_O_3_ catalysts derived from both cp- and ht-LNO precursors, both materials were tested under methane dry reforming conditions for 100 hours at 650 °C (CH_4_:CO_2_ 1:1, GHSV = 100 L/g·h). After testing, we found that the catalyst derived from the ht-LNO precursor showed an enormous amount of carbon accumulation in the form of multi-walled carbon nanotubes (MWCNTs) while the catalyst derived from the cp-LNO was found to be carbon-free when tested under identical conditions (Fig. S8). MWCNT growth is facilitated by the “tip-growth” mechanism and is enabled due to weak bonding between the Ni catalyst particle and the La_2_O_3_ support. Such weak catalyst bonding allows the growing MWCNT to dislodge and lift the Ni particle from the support. Carbon growth ultimately leads to catalyst deactivation by blocking active sites and clogging the pores of the support. The fact that the “tip-growth” mechanism was entirely restricted on catalysts derived from cp-LNO indicates that Ni particles reduced from this precursor via the 3-step mechanism yields Ni crystals with a markedly stronger bonding to the La_2_O_3_.

A possible drawback of solid phase crystallization technique for the synthesis of supported nanoparticle catalysts is the possibility of exsolving Ni particles completely inside the support. Intelligent catalyst design dictates that Ni nanoparticles should preferably have part of its surface exposed outside of the support since crystals buried within the La_2_O_3_ particle may not contribute catalytically. In principle, small deviations in surface tension, and strain should help to determine whether the Ni particle has an exposed surface or not^32^.

**Figure 4:**
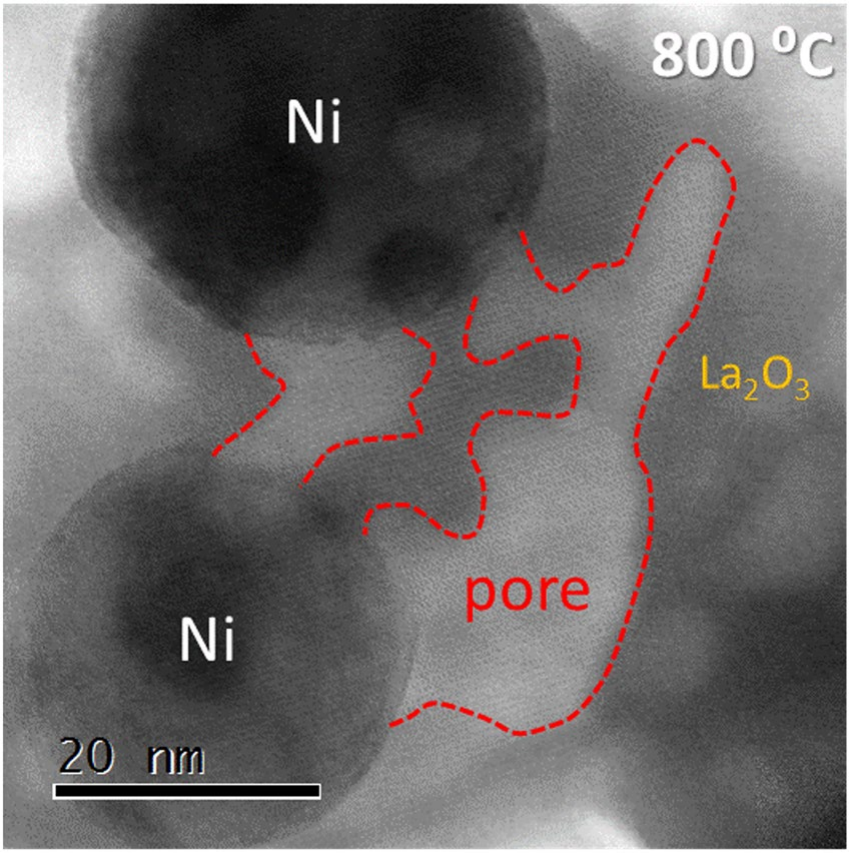
Bright-field *in-situ* STEM image of cp-LNO at 800 °C in H_2_ atmosphere (250 mbar). Dashed line represents the boundary of internal pores formed during the crystallization process. Similar porous structures were observed for ht-LNO after reduction.

We observed a 50% increase in Brunauer-Emmet-Teller (BET) surface area after the solid-phase crystallization process (from 10 cm^2^ to 15 cm^2^ for both ht-LNO and cp-LNO. *In-situ* STEM imaging of the material after complete reduction (800 °C), in Fig. 4, reveals the reason for this: the materials form a porous network within the support during the crystallization process. Similar behavior was observed for cp-LNO. Pores and Ni crystals are differentiated here by STEM-HAADF (not shown) since the former gives dark contrast and the latter bright intensity. This network leaves the reduced La_2_O_3_ support with a higher porosity than the LaNiO_3_ precursor it formed from. Such a network is likely the result of differences in molar volume during reduction and the high degree of deformation and internal strain due to the co-existing phases present during the crystallization steps.

This work highlights the importance of understanding the defect structure of precursors used to prepare catalysts by solid-phase crystallization. Subtle differences in the synthesis method can give rise to large differences in the extended defect structures and our results suggest that this can dramatically alter the crystallization path and subsequent performance of the catalyst. Furthermore, our work suggests that the development of more precise techniques to understand and control defect formation in oxide ceramics could open up the field of “defect engineering” for catalytic materials.

## Experimental

The cell used to perform the *in-situ* synchrotron radiation XRD is described by P.J. Chupas^33^. The DFT calculations are carried out using the Vienna Ab Initio Package^34^ with projector augmented-wave pseudopotentials^35^ to obtain the reaction free energies. The electronic exchange-correlation interaction is described using the metaGGA-MS2 density functional^29^ which captures the nonlocal nature of electronic exchange by including the electronic kinetic energy. The cutoff energy is 500 eV, and the reciprocal *k*-grid has a spacing of <~0.25 Å^−1^. The calculation convergence thresholds for energy and force are 10^−6^ eV and 10^−3^ eV Å^−1^, respectively. The crystal structures for all the involved oxides in Equations 1–5 have been experimentally characterized are reported in the *Inorganic Crystal Structures Database* [36]; these structures are used as the initial structures for the DFT calculations.

Scanning transmission electron microscopy (STEM) and electron energy loss spectroscopy (EELS) measurements were carried out using a FEI Titan G2 80-200 (S)TEM ChemiSTEM instrument operated at 200 keV with a convergence angle of 21 mrad at the University of Manchester. High resolution STEM imaging and spectrum imaging were performed with a probe current of 90pA and 180 pA, respectively. The Titan instrument is equipped with the FEI SuperX Quad SDD array system (~0.7 sR collection angle) and a Quantum ER dual EELS spectrometer. The gas-heating e-Cell system used for this study was from Protochips. Although, this atmosphere holder has a customized low penumbra geometry titanium lid^26^, for EDXS acquisition with the gas-heating cell, only two of the EDXS detectors were used and the specimen holder was tilted at 25° to reduce the shadowing of the cell^36, 37^. For EDXS in the vacuum-heating cell, no tilting is necessary and therefore all four EDXS detector can be used. Data was recorded using Gatan DigitalMicrograph software, post processing of STEM-EDX spectrum images was carried out using DigitalMicrograph and Hyperspy software. The e-Cell MEMS chips used to create the operando environment were of a pair of 300 µm thick Si wafers; with each having a lithographically fabricated, 3000 × 300 µm, electron transparent SiNx window. The upper chip allows heating and has a SiNx window thickness of 30 nm, while the lower window is 50 nm thick. Spacers deposited onto the chips created a nominal vertical separation between the windows of approximately 5 µm. During STEM measurements the e-Cell was completely filled with 3%H_2_/N_2_ at nominally 250 mbar bar pressure while the temperature was controlled by on-chip pre-calibrated heater elements controlled by an external computer system. The reduction of the cp-LNO sample was carried out in the gas-heating cell described above while the ht-LNO sample was reduced in a similar vacuum-heating cell. Our study revealed that the same pathway is observed regardless of whether the gas-heating or vacuum-heating cell was used, indicating that the vacuum environment in the heating cell was of sufficiently reducing character. While small shifts in temperature (50–100 degrees) were observed between these cells, these shifts do not impact the primary findings of this report.

## Electronic supplementary material

Supplementary info
