# Supplementary material for: Role of 2D and 3D defects on the reduction of LaNiO3 nanoparticles for catalysis
Source: Sci Rep. 2017 Aug 30;7:10080. doi: 10.1038/s41598-017-10703-5 (PMC5577106; doi:10.1038/s41598-017-10703-5)
Supplement: Supplementary file 1 — Supplementary info [file 41598_2017_10703_MOESM1_ESM.pdf]

Supporting Information for:

Role of 2D and 3D defects on the reduction of $LaNiO_3$ nanoparticles for catalysis

Sarika Singh[†], Eric Prestat[#], Liang-Feng Huang[+], James M. Rondinelli[+], Sarah J. Haigh[#], Brian A. Rosen[†]*

[†] Department of Materials Science and Engineering, Tel Aviv University, 55 Haim Levanon Street, Ramat Aviv Israel, 69987001, Israel

[#]School of Materials, The University of Manchester, Oxford Road, M13 9PL, UK

[+] Department of Materials Science and Engineering, Northwestern University, Evanston Illinois 60208-3108, USA

***barosen@post.tau.ac.il**

Table S1: Summary of major reports of reduction mechanism and synthesis method used

#		Mechanism	Synthesis method	Materia l	Size of $LaNiO_3$/ Ni (nm)	Ref.
1.	Two step process	$2LaNiO_3+H_2 \rightarrow La_2Ni_2O_5+H_2O$<br>$La_2Ni_2O_5+2H_2 \rightarrow La_2O_3+2Ni^0+2H_2O$	Sol–gel method in propionic acid	$LaNiO_3$	$LaNiO_3$= 22 nm	[1]
2.	Two step process	$2LaNiO_3+2H_2 \rightarrow La_2NiO_4+Ni^0+2H_2O$<br>$La_2NiO_4+H_2 \rightarrow La_2O_3+Ni^0+H_2O$	Co-precipitation oxidation hydrothermal method	$LaNiO_3$	Ni=10 nm	[2]
3.	Three step process	$4LaNiO_3+2H_2 \rightarrow La_4Ni_3O_{10}+Ni^0+2H_2O$<br>$La_4Ni_3O_{10}+3H_2 \rightarrow La_2NiO_4+2Ni^0+La_2O_3+3H_2O$<br>$La_2NiO_4+H_2 \rightarrow Ni^0+La_2O_3+H_2O$	Self-combustion method	$LaNiO_3$	Ni=15 nm	[3]
4.	Three step process	$4LaNiO_3+2H_2 \rightarrow La_4Ni_3O_{10}+Ni^0+2H_2O$<br>$La_4Ni_3O_{10}+3H_2 \rightarrow La_2NiO_4+2Ni^0+La_2O_3+3H_2O$<br>$La_2NiO_4+H_2 \rightarrow Ni^0+La_2O_3+H_2O$	Citrate method	$LaNiO_3$/ SBA-15	Ni=7.7 nm	[4]
5.	Two step	$LaNiO_3+H_2 \rightarrow LaNiO_{2.7}+NiO+H_2$	Sol–gel	$LaNiO_3$	Ni=9nm	[5]

	process	$LaNiO_{2.7}+H_2 \rightarrow La_2O_3+Ni^0+H_2$	method			
6.	Two step process	-	Citrate sol gel method	$LaNiO_3$ @SiO_2	$LaNiO_3$ = 20nm	[6]
7.	Two step process	-	Spray pyrolysis	$LaNiO_3$	$LaNiO_3$ 150–500 nm,<br>Ni=38 nm	[7]
8.	Two step process	-	Citrate complex	$LaNiO_3$ -SBA	Ni=47 nm	[8]
9.	Two step process	-	Citrate method	$LaNiO_3$	$LaNiO_3$= 50-100nm<br>Ni=13 nm	[9]
	Two step process	-	Pechini method	$LaNiO_3$	$LaNiO_3$= 30-100nm<br>Ni=16nm	
	Two step process	-	Propionate method	$LaNiO_3$	$LaNiO_3$= 30-100nm<br>Ni=14nm	
10.	Two step process	-	Hydrothermal synthesis	$LaNiO_3$	$LaNiO_3$= 40–80 nm<br>Ni=19nm	[10]
	Two step process	-	Combustion method	$LaNiO_3$	$LaNiO_3$= 40–80 nm<br>Ni=42nm	
	Two step process	-	Spray pyrolysis	$LaNiO_3$	$LaNiO_3$= 20–40 nm<br>Ni= 69nm	
	Two step process	-	Spray pyrolysis-combustion	$LaNiO_3$	$LaNiO_3$=300nm-1μmNi=30nm	
11.	Three step process	$LaNiO_3+0{:}25H_2 \rightarrow LaNiO_{2:75}+0{:}25H_2O$<br>$4LaNiO_{2:75}+H_2 \rightarrow La_4Ni_3O_{10}+Ni^0+H_2O$<br>$La_4Ni_3O_{10}+4H_2 \rightarrow 2La_2O_3+3Ni^0+4H_2O$	Citrate sol–gel method	bulk $LaNiO_3$	-	[11]
12.	Two step process	$2LaNiO_3+H_2 \rightarrow LaNi_2O_5+H_2O$<br>$La_2Ni_2O_5+2H_2 \rightarrow La_2O_3+2Ni^0+2H_2O$	Sol-gel method in propionic acid	$LaNiO_3$	-	[12]
13.	Two step process	$2LaNiO_3+H_2 \rightarrow La_2Ni_2O_5+H_2O$<br>$La_2Ni_2O_5+2H_2 \rightarrow La_2O_3+2Ni^0+2H_2O$	Sol-gel method in propionic acid	$LaNiO_3$	$LaNiO_3$ =22 nm	[13]
14.	Two step process	$2LaNiO_3+H_2 \rightarrow La_2Ni_2O_5+H_2O$<br>$La_2Ni_2O_5+2H_2 \rightarrow La_2O_3+2Ni^0+2H_2O$	Proteic gel method, collagen powder as chelating agent	$LaNiO_3$	$LaNiO_3$= 13nm	[14]
15.	Three step process	$4LaNiO_3+2H_2 \rightarrow La_4Ni_3O_{10}+Ni^0+2H_2O$<br>$La_4Ni_3O_{10}+3H_2 \rightarrow La_2NiO_4+2Ni^0+La_2O_3+3H_2O$	Explosion method	$LaNiO_3$	Ni=70–80 nm	[15]

		$La_2NiO_4+H_2 \rightarrow Ni+La_2O_3+H_2O$				
16.	Three step process	$4LaNiO_3+2H_2 \rightarrow La_4Ni_3O_{10}+Ni^0 +2H_2O$<br>$La_4Ni_3O_{10}+3H_2 \rightarrow La_2NiO_4+2Ni^0 +La_2O_3+3H_2O$<br>$La_2NiO_4+H_2 \rightarrow Ni^0+La_2O_3+H_2O$	Self-combustion method	$LaNiO_3$	Ni=15 nm	[16]
17.	Two Step Process	$2LaNiO_3+2H_2 \rightarrow La_2NiO_4+Ni^0+ 2H_2O$<br>$4LaNiO_3+H_2 \rightarrow La_2Ni_2O_5+2Ni^0+ La_2O_3+4H_2O$	Co-precipitation method using tetraethyl ammonium hydroxide	$LaNiO_3$	-	[17]

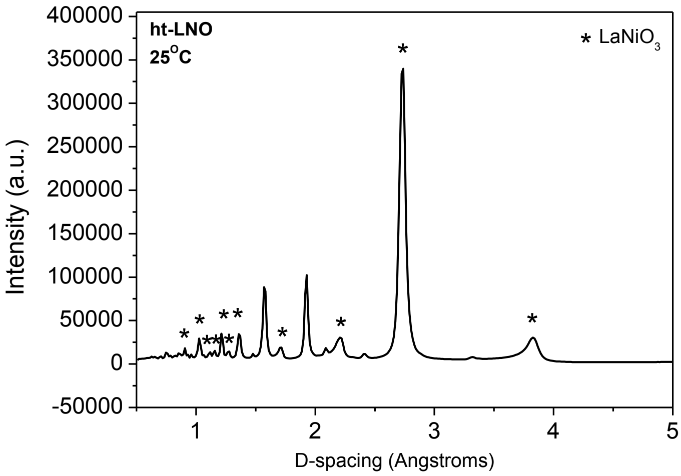

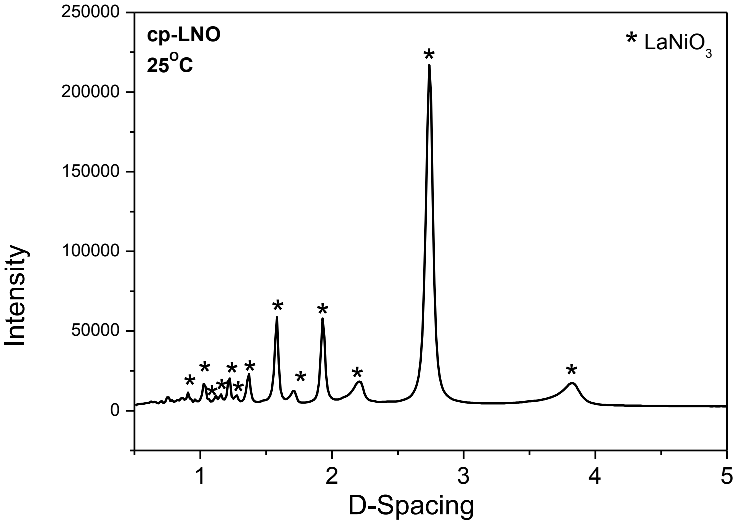

Figure S1. XRD patterns for ht-LNO and cp-LNO at room temperature

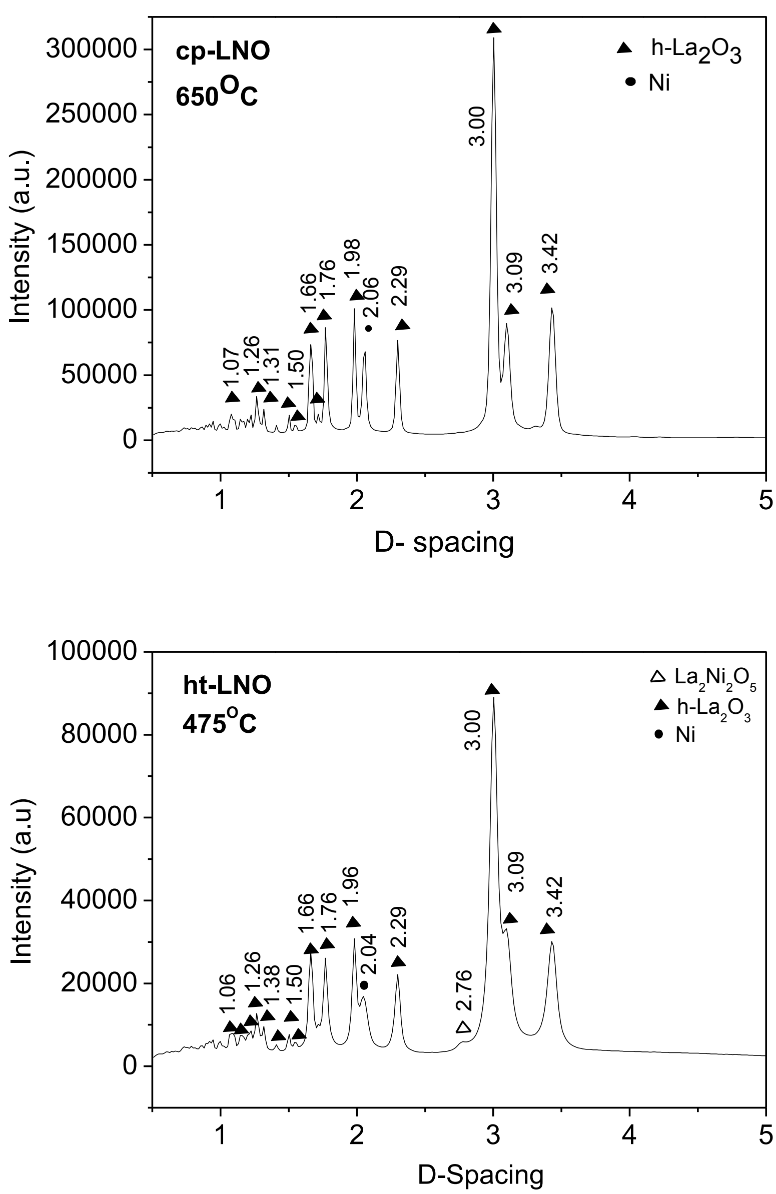

Figure S2**:** XRD-patters of cp- and ht-LNO at the lowest temperature where the original LNO structure is completely destroyed.

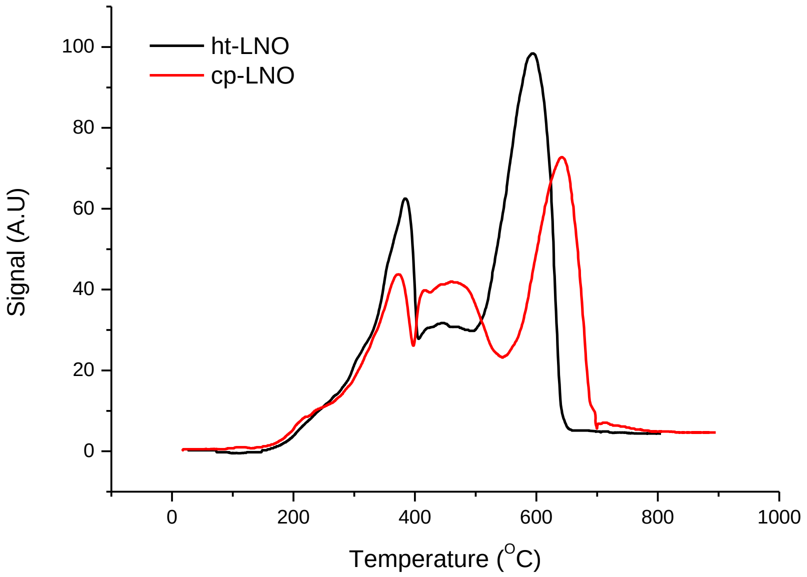

Figure S3. Temperature programmed reduction of ht- and cp-LNO using 5% H_2/N_2 and a temperature ramp of 10°C/min. In both cases, exactly 30 mg of LNO was analyzed.

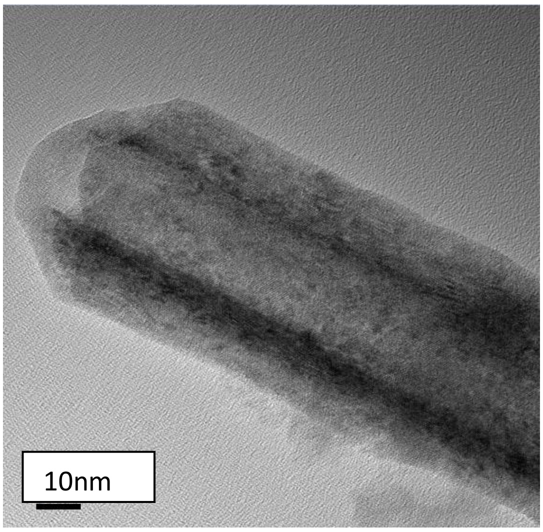

Figure S4. TEM image of LaOH precursor to the cp-LNO structure. Calcination of LaOH and NiOH at 800°C gave rise to the $LaNiO_3$ structure

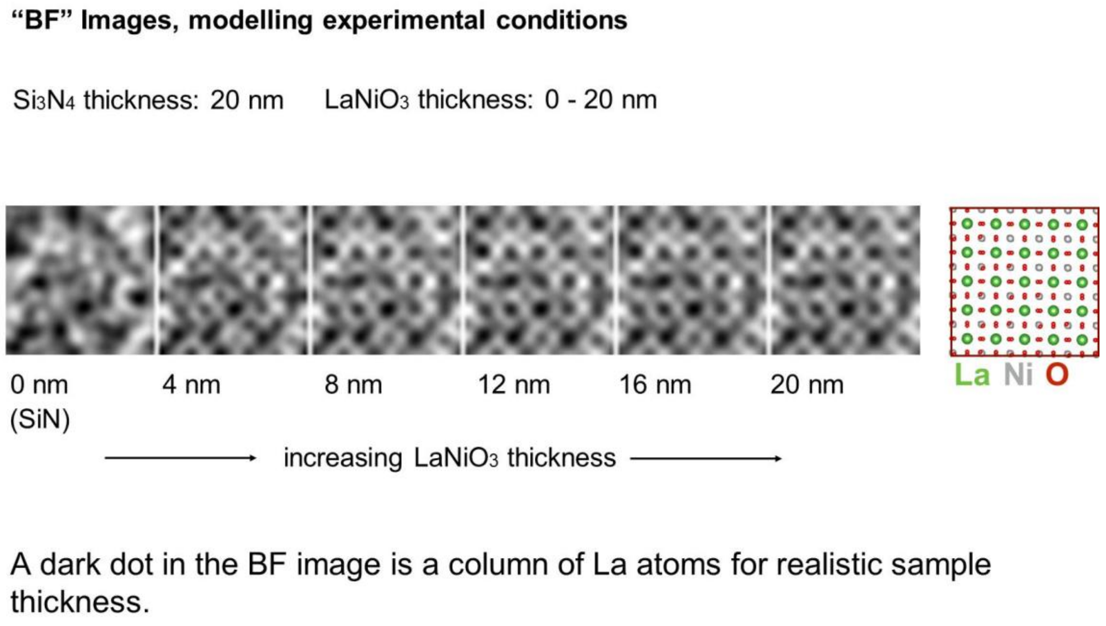

Figure S5. BF STEM simulations to determine the contrast effects in in-situ STEM imaging of $LaNiO_3$ samples of varying thickness supported on a silicon nitride grid.

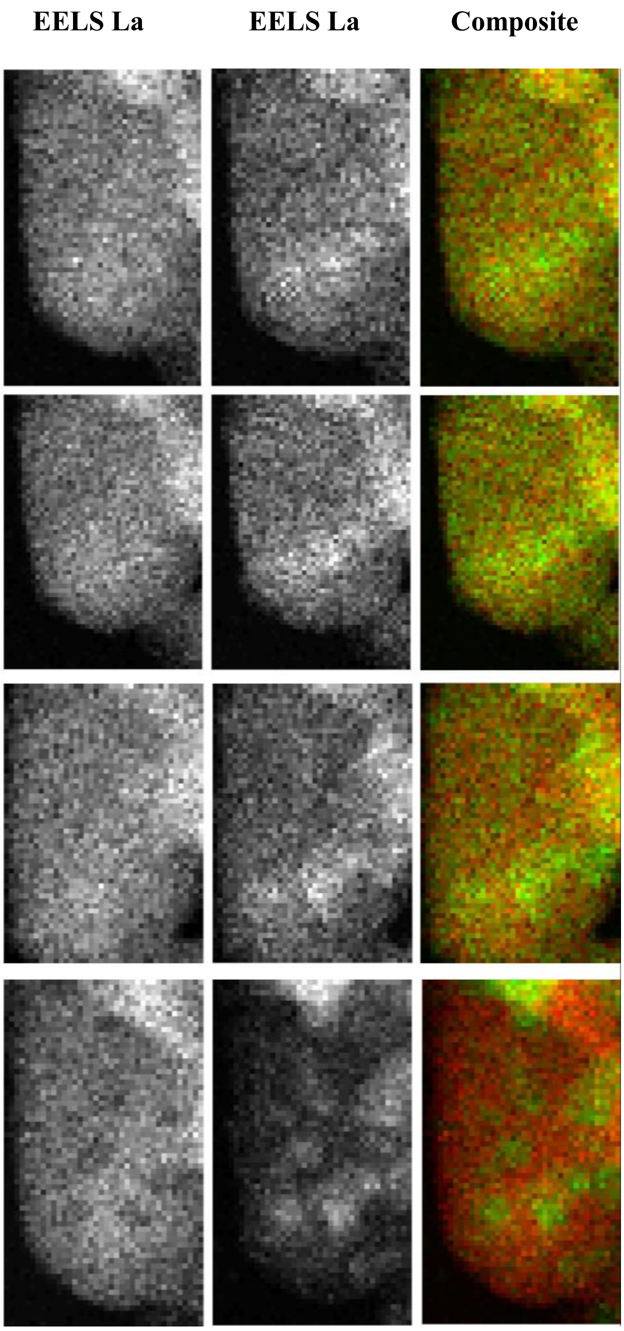

Figure S6: Individual and composite EELS maps for Ni and Li from ht-LNO at 350, 450, 600, and 700°C (from top to bottom).

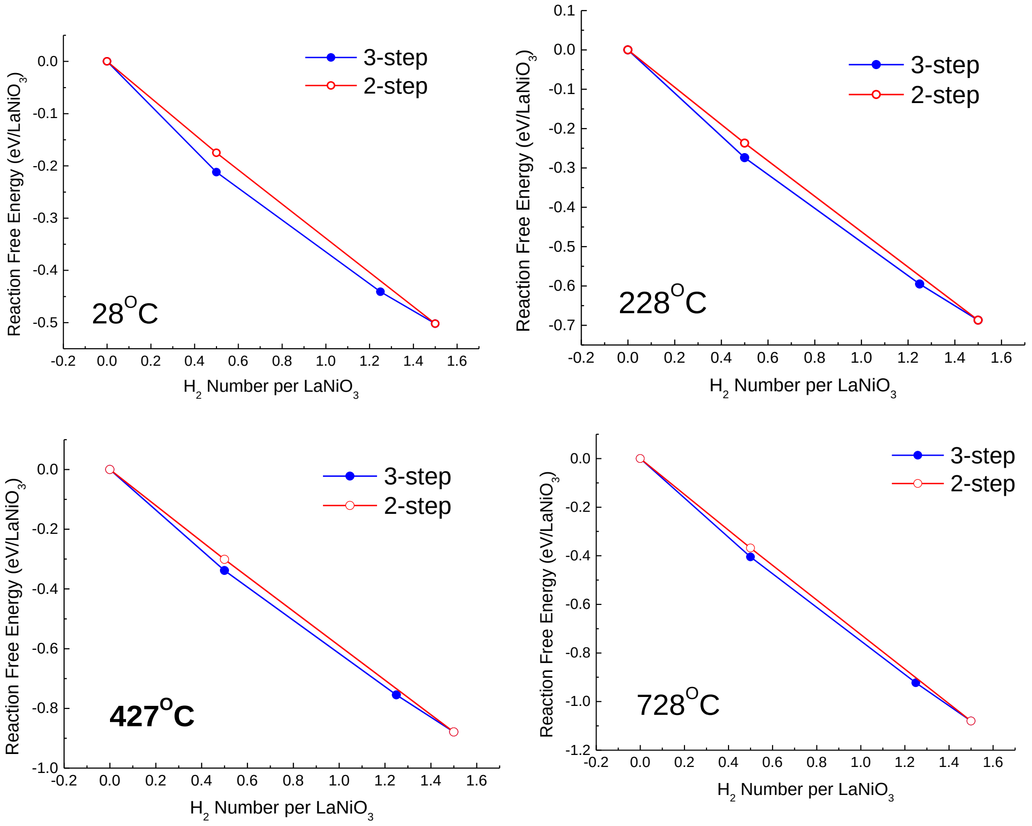

Figure S7. MS2 density functional theory calculation showing the reaction free energy for the 3- and 2-step crystallization paths at 300, 500, 700, and 900K

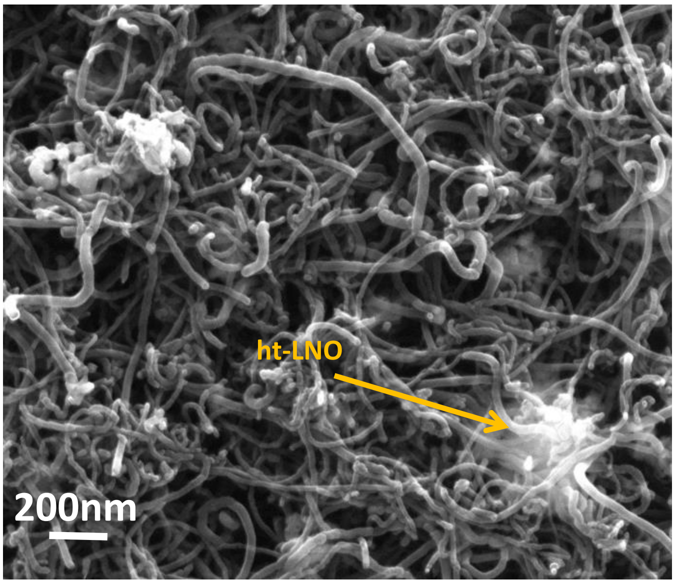

Figure S8. SEM image of severe multi-walled carbon nanotube (MWCNT) growth on ht-LNO derived Ni/$LaNiO_3$ after operating under methane dry reforming conditions.

Calculation of Formation Free Energies and Reaction Free Energies of La-Ni-O compounds via DFT

(I) Reduction paths and energies for $LaNiO_3$

(A) 3-step reactions

(1) $4*LaNiO_3 + 2*H_2 \rightarrow La_4Ni_3O_{10} + Ni + 2*H_2O$

(2) $La_4Ni_3O_{10} + 3*H_2 \rightarrow La_2NiO_4 + 2*Ni + La_2O_3 + 3*H_2O$

(3) $La_2NiO_4 + H_2 \rightarrow Ni + La_2O_3 + H_2O$

(B) 2-step reactions

(1) $2*LaNiO_3 + H_2 \rightarrow La_2Ni_2O_5 + H_2O$

(2) $La_2Ni_2O_5 + 2*H_2 \rightarrow 2*Ni + La_2O_3 + 2*H_2O$

(II) Formation Energy and Formation Free Energy

To define the formation energy of La-Ni-O compounds, La_2O_3, Ni, O_2 (1 atm), and H_2 (1 atm) are used as references. Then, we have:

$LaNiO_3 = 1/2*La_2O_3 + Ni + 3/4*O_2$

$La_2NiO_4 = La_2O_3 + Ni + 1/2*O_2$

$La_3Ni_2O_7 = 3/2*La_2O_3 + 2*Ni + 5/4*O_2$

$La_4Ni_3O_{10} = 2*La_2O_3 + 3*Ni + 2*O_2$

$La_2Ni_2O_5 = La_2O_3 + 2*Ni + O_2$

The formation energies (***E_f* per formula unit**) are calculated as:

$E_f(LaNiO_3) = E_{DFT}(LaNiO_3) - 1/2*E_{DFT}(La_2O_3) - E_{DFT}(Ni) - 3/4*E_{DFT}(O_2)$
$E_f(La_2NiO_4) = E_{DFT}(La_2NiO_4) - E_{DFT}(La_2O_3) - E_{DFT}(Ni) - 1/2*E_{DFT}(O_2)$
$E_f(La_3Ni_2O_7) = E_{DFT}(La_3Ni_2O_7) - 3/2*E_{DFT}(La_2O_3) - 2*E_{DFT}(Ni) - 5/4*E_{DFT}(O_2)$
$E_f(La_4Ni_3O_{10}) = E_{DFT}(La_4Ni_3O_{10}) - 2*E_{DFT}(La_2O_3) - 3*E_{DFT}(Ni) - 2*E_{DFT}(O_2)$
$E_f(La_2Ni_2O_5) = E_{DFT}(La_2Ni_2O_5) - E_{DFT}(La_2O_3) - 2*E_{DFT}(Ni) - E_{DFT}(O_2)$

The formation free energies (**G_f per formula unit**) are:
$G_f(LaNiO_3) = E_f(LaNiO_3) - 3/4*\Delta_T G(O_2)$
$G_f(La_2NiO_4) = E_f(La_2NiO_4) - 1/2*\Delta_T G(O_2)$
$G_f(La_3Ni_2O_7) = E_f(La_3Ni_2O_7) - 5/4*\Delta_T G(O_2)$
$G_f(La_4Ni_3O_{10}) = E_f(La_4Ni_3O_{10}) - 2*\Delta_T G(O_2)$
$G_f(La_2Ni_2O_5) = E_f(La_2Ni_2O_5) - \Delta_T G(O_2)$

where the vibrational free energies of the solids and molecules are omitted, and only translational and rotational free energies of O_2 (1 atm) gas are considered. The temperature dependent part in O_2 free energy [$\Delta_T G(O_2)$] and the formation free energy of H_2O vapour are obtained from the NIST-JANAF database [18]:

The partial pressure of the H_2 gas and H_2O vapour is considered in the chemical potential as:

$$\mu_P = \mu_{P_0} + k_B T * \ln(\frac{P}{P_0})$$

while, the reaction free energies are independent of partial pressure (shown below).

Table S2. Formation free energies of La-Ni-O compounds (eV per formula unit) using DFT-MS2.

T (K)	$LaNiO_3$	La_2NiO_4	$La_3Ni_2O_7$	$La_4Ni_3O_{10}$	$La_2Ni_2O_5$
0	-3.461	-2.398	-5.941	-9.409	-4.630
100	-3.348	-2.323	-5.753	-9.109	-4.480
200	-3.205	-2.228	-5.515	-8.727	-4.289
300	-3.050	-2.124	-5.256	-8.313	-4.082
400	-2.887	-2.016	-4.985	-7.879	-3.865
500	-2.718	-1.903	-4.702	-7.427	-3.639
600	-2.544	-1.787	-4.413	-6.965	-3.408
700	-2.367	-1.669	-4.117	-6.491	-3.171
800	-2.184	-1.547	-3.812	-6.003	-2.927
900	-1.998	-1.423	-3.503	-5.509	-2.680
1000	-1.812	-1.299	-3.193	-5.011	-2.431

(III) Reaction Free Energy (eV per $LaNiO_3$)

For the 3-step reaction pathway
$\Delta G1 = 1/4*G_f(La_4Ni_3O_{10}) + 1/4*G_f(Ni) - G_f(LaNiO_3) + 1/2*[\mu(H_2O) - \mu(H_2)]$
$\Delta G2 = 1/4*G_f(La_2NiO_4) + 1/2*G_f(Ni) + 1/4*G_f(La_2O_3) - 1/4*G_f(La_4Ni_3O_{10}) + 3/4*[\mu(H_2O) - \mu(H2)]$
$\Delta G3 = 1/4*G_f(Ni) + 1/4*G_f(La_2O_3) - 1/4*G_f(La_2NiO_4) + 1/4*[\mu(H_2O) - \mu(H_2)]$

For the 2-step reaction pathway
$\Delta G1 = 1/2*G_f(La_2Ni_2O_5) - G_f(LaNiO_3) + 1/2*[\mu(H_2O) - \mu(H_2)]$
$\Delta G2 = G_f(Ni) + 1/2*G_f(La_2O_3) - 1/2*G_f(La_2Ni_2O_5) + [\mu(H_2O) - \mu(H_2)]$

According to the expressions for the reaction free energies, it can be shown that they are independent of partial pressure; therefore, only temperature variations are considered.

Table S3. Reaction free energies (eV per $LaNiO_3$)

	3-Step			2-Step	
T (K)	1	2	3	1	2
0	-0.129	-0.104	-0.020	-0.092	-0.161
100	-0.155	-0.142	-0.032	-0.118	-0.212
200	-0.183	-0.184	-0.046	-0.145	-0.267
300	-0.212	-0.229	-0.061	-0.175	-0.327
400	-0.243	-0.275	-0.076	-0.206	-0.389
500	-0.274	-0.321	-0.092	-0.237	-0.450
600	-0.306	-0.369	-0.108	-0.269	-0.514
700	-0.338	-0.417	-0.124	-0.301	-0.578
800	-0.372	-0.468	-0.141	-0.334	-0.645
900	-0.405	-0.518	-0.157	-0.368	-0.713
1000	-0.439	-0.569	-0.174	-0.402	-0.780

REFERENCES

[1] Moradi GR, Rahmanzadeh M, Khosravian F. The effects of partial substitution of Ni by Zn in LaNiO3 perovskite catalyst for methane dry reforming. Journal of CO2 Utilization 2014;6:7-11.
[2] Lin K-H, Wang C-B, Chien S-H. Catalytic performance of steam reforming of ethanol at low temperature over LaNiO3 perovskite. International Journal of Hydrogen Energy 2013;38:3226-32.
[3] Sierra Gallego G, Batiot-Dupeyrat C, Barrault J, Mondragón F. Dual Active-Site Mechanism for Dry Methane Reforming over Ni/La2O3 Produced from LaNiO3 Perovskite. Ind Eng Chem Res 2008;47:9272-8.
[4] Wang N, Yu X, Wang Y, Chu W, Liu M. A comparison study on methane dry reforming with carbon dioxide over LaNiO3 perovskite catalysts supported on mesoporous SBA-15, MCM-41 and silica carrier. Catalysis Today 2013;212:98-107.
[5] Jia L, Li J, Fang W. Effect of H2/CO2 mixture gas treatment temperature on the activity of LaNiO3 catalyst for hydrogen production from formaldehyde aqueous solution under visible light. Journal of Alloys and Compounds 2010;489:L13-L6.
[6] Zheng X, Tan S, Dong L, Li S, Chen H. LaNiO3@SiO2 core–shell nano-particles for the dry reforming of CH4 in the dielectric barrier discharge plasma. International Journal of Hydrogen Energy 2014;39:11360-7.
[7] Pereñíguez R, González-DelaCruz VM, Holgado JP, Caballero A. Synthesis and characterization of a LaNiO3 perovskite as precursor for methane reforming reactions catalysts. Applied Catalysis B: Environmental 2010;93:346-53.
[8] Nair MM, Kaliaguine S, Kleitz F. Nanocast LaNiO3 Perovskites as Precursors for the Preparation of Coke-Resistant Dry Reforming Catalysts. ACS Catalysis 2014;4:3837-46.
[9] Kuras M, Roucou R, Petit C. Studies of LaNiO3 used as a precursor for catalytic carbon nanotubes growth. Journal of Molecular Catalysis A: Chemical 2007;265:209-17.
[10] Pereñiguez R, Gonzalez-delaCruz VM, Caballero A, Holgado JP. LaNiO3 as a precursor of Ni/La2O3 for CO2 reforming of CH4: Effect of the presence of an amorphous NiO phase. Applied Catalysis B: Environmental 2012;123–124:324-32.

[11] Rivas I, Alvarez J, Pietri E, Pérez-Zurita MJ, Goldwasser MR. Perovskite-type oxides in methane dry reforming: Effect of their incorporation into a mesoporous SBA-15 silica-host. Catalysis Today 2010;149:388-93.
[12] Moradi GR, Rahmanzadeh M. The influence of partial substitution of alkaline earth with La in the LaNiO3 perovskite catalyst. Catalysis Communications 2012;26:169-72.
[13] Moradi GR, Khosravian F, Rahmanzadeh M. Effects of Partial Substitution of Ni by Cu in LaNiO3 Perovskite Catalyst for Dry Methane Reforming. Chinese Journal of Catalysis 2012;33:797-801.
[14] Santos JC, Souza MJB, Ruiz JAC, Melo DMA, Mesquita ME, Pedrosa AMG. Synthesis of LaNiO3 perovskite by the modified proteic gel method and study of catalytic properties in the syngas production. Journal of the Brazilian Chemical Society 2012;23:1858-62.
[15] Batiot-Dupeyrat C, Valderrama G, Meneses A, Martinez F, Barrault J, Tatibouët JM. Pulse study of CO2 reforming of methane over LaNiO3. Applied Catalysis A: General 2003;248:143-51.
[16] Gallego GS, Batiot-Dupeyrat C, Barrault J, Florez E, Mondragón F. Dry reforming of methane over LaNi1−yByO3±δ (B = Mg, Co) perovskites used as catalyst precursor. Applied Catalysis A: General 2008;334:251-8.
[17] Crespin M, Levitz P, Gatineau L. Reduced forms of LaNiO3 perovskite. Part 1.- Evidence for new phases: La2Ni2O5 and LaNiO2. Journal of the Chemical Society, Faraday Transactions 2: Molecular and Chemical Physics 1983;79:1181-94.

[18] M. W. Chase, Jr. “NIST-JANAF Thermochemical Tables” (National Institute of Standards and Technology, Gaithersburg, Maryland, 1998).
